# Technology enabled home-based cardiac rehabilitation among women with cardiovascular disease: A longitudinal cohort study

**DOI:** 10.1016/j.ijcrp.2023.200226

**Published:** 2023-11-22

**Authors:** Michael Najem, Mark Duggan, Rebecca Gambatese, Rebecca Hill, Su-Jau Yang, Columbus Batiste, Tadashi Funahashi, Chileshe Nkonde-Price

**Affiliations:** a Department of Clinical Science, Kaiser Permanente Bernard J. Tyson School of Medicine, Pasadena, CA, USA; b Office of Research and Scholarship, Kaiser Permanente Bernard J. Tyson School of Medicine, Pasadena, CA, USA; c Department of Research & Evaluation, Kaiser Permanente Southern California, Pasadena, CA, USA; d Department of Cardiology, Southern California Permanente Medical Group, USA; e Kaiser Permanente Center for Health Innovation, Tustin, CA, USA; f Department of Orthopedic Surgery, Southern California Permanente Medical Group, USA

## Abstract

Technology-enabled home-based cardiac rehabilitation (HBCR) is an emerging alternative to traditional center-based cardiac rehabilitation (CBCR), but little is known about outcomes in women. We analyzed 753 diverse and medically complex women who participated in HBCR and CBCR within an integrated health system and found both groups had similar clinical outcomes. Results suggest HBCR is a viable alternative to CBCR among women, including women with multiple comorbidities.

## Funding source

None.

Center based cardiac rehabilitation (CBCR) performed in the hospital-based setting is a well-studied effective intervention known to improve health outcomes in patients with cardiovascular disease (CVD) [[1], [2], [3], [4]]. Guidelines recommend CBCR in patients post myocardial infarction (MI) [[5], [6], [7]], post cardiac surgery [8], in patients with congestive heart failure [9,10], and patients with peripheral artery disease [11]. Despite this recommendation, studies have shown that >80 % of eligible patients in the United States (US) do not participate in CBCR [12], with the lowest participation rates in women, ethnic minorities and patients with multiple co-morbidities [3,4,[13], [14], [15]].

CR performed in the non-hospital setting such as home-based CR (HBCR) is an alternative to CBCR and has been shown to have similar outcomes in randomized controlled trials (RCT) [1]. Additionally, the Cochrane Collaboration has conducted 3 meta-analyses that have combined RCTs of HBCR vs CBCR and consistently found that HBCR and CBCR are associated with similar outcomes in the selected lower risk patients enrolled in these trials [1]. Unfortunately, the benefits of HBCR for women are unclear because the majority of HBCR vs CBCR RCTs included primarily men [1,12].

To address this knowledge gap, we studied 753 women who participated in HBCR or CBCR between April 1, 2018 and April 30, 2019 within the Kaiser Permanente Southern California (KPSC) integrated health system and had ≥12 months continuous KPSC membership before and after CR enrollment.

Details of the KPSC HBCR Program have been previously described [16,17]. Briefly, patients with clinical indication for CR (acute myocardial infarction (MI), stable angina, chronic heart failure (HF), elective percutaneous coronary intervention (PCI), coronary artery bypass graft (CABG), and valve repair or replacement surgery) were referred to the program at the discretion of the treating cardiologist. HBCR was delivered through mobile phone application linked to a wearable smartwatch and involved an 8-week comprehensive multidisciplinary program that consisted of (1) unsupervised exercise monitored by a smartwatch, (2) weekly CR nurse telephone support, and (3) virtual and in-person health education. This comprehensive structure was similar to the majority of RCTs included in the Cochrane HBCR vs. CBCR meta-analysis [1]. CBCR involved supervised exercise sessions (36 sessions of 30-min duration) performed at CR centers accredited by the American Association of Cardiovascular and Pulmonary Rehabilitation. Attendance of ≥1 session in both programs defined participation and served as the primary exposure.

The primary outcome was 12-month all-cause hospitalization following CR participation. Data on hospitalizations that occurred within the 12-month period following enrollment in CR were extracted from the medical record and billing claims from outside services. The principal diagnosis for each hospitalization was assessed using the primary ICD-10 code, which reflects the main reason for admission. The primary reason for hospitalization was divided into all cause (all ICD 10 codes) or cardiovascular (ICD 10 Codes—I00-I99).

To assess baseline differences between HBCR and CBCR participants, we used chi-squared tests for categorical variables and independent samples t-tests for continuous variables. Due to differences between these two groups, we used propensity score weighting to balance baseline characteristics, estimating the average treatment effect (ATE) among those who participated in home-based cardiac rehab on the following covariates: age, race/ethnicity, rehabilitation referral reason (CABG, CHF, valve surgery, or coronary artery disease), smoking status, BMI, prior inpatient stay, Charlson Comorbidity index score, and select comorbid conditions (atrial fibrillation, hypertension, prior myocardial infarction, and prior stroke). Propensity score weights were estimated using gradient boosted models via the twang package in R(v2.5) [18]. Standardized differences were calculated to compare baseline characteristics before and after weighting. The weighted standardized differences of all the baseline characteristics were less than 0.1, indicating good balance between rehabilitation programs [19]. We then extracted the weights and fit a weighted multivariable logistic regression model to estimate the effect of home-based rehab compared to center-based rehab on 12-month all-cause hospitalization, adjusting for demographics, comorbid conditions, referral reason, prior inpatient stay, and treatment weights. All statistical analyses were performed using R Statistical Software (v4.0.5)^20^, with statistical significance set at p < 0.05. The present study was approved by the KPSC institutional review board. A waiver of informed consent was obtained because of the nature of the study.

**Fig. 1:**
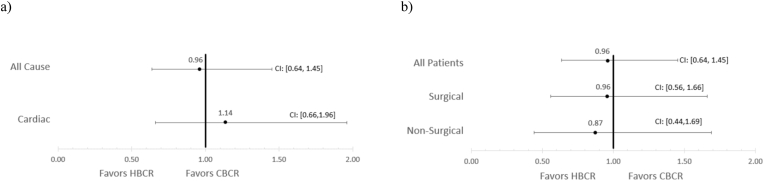
Hospitalization events for HBCR and CBCR after propensity sore weighting a) Propensity-weighted odds ratios of all-cause and cardiovascular disease-specific hospitalization rates at 12 months following participation in HBCR and CBCR b) Propensity-weighted odds ratios of all-cause hospitalizations at 12 months following participation in HBCR and CBCR stratified by referral reason to cardiac rehabilitation: *All Patients*-entire cohort; *Surgical*-heart valve surgery and coronary artery bypass grafting; *Non-Surgical*-coronary artery disease (angina, percutaneous coronary intervention, myocardial infarction) and heart failure.

Of 753 women included (mean age, 67.6 years; 44.8 % racial and ethnic minority groups; 50.2 % medically complex), 382 (50.7 %) participated in HBCR (Table 1). After propensity score weighting, there were similar 12-month all-cause hospitalization rates between women who participated in HBCR compared with those who participated in CBCR (65 women (17 %) vs. 64 women (18.1 %); OR, 0.96; 95 % CI, 0.64–1.45) (Fig. 1). 12-month hospitalization rates due to cardiovascular disease were also similar between HBCR and CBCR (39 women (10.2 %) vs. 32 women (9.4 %); OR, 1.14; 95 % CI, 0.66–1.96) (Fig. 1). Patients were further stratified by CR referral reason into surgical (valve surgery and CABG) and non-surgical (coronary artery disease (MI, angina, and PCI) and HF) subgroups. Similar 12-month hospitalization rates between HBCR and CBCR patients were found in the surgical (OR, 0.96; 95 % CI, 0.56–1.66) and non-surgical (OR, 0.87; 95 % CI, 0.44–1.69) groups (Fig. 1).

**Table 1:** **Propensity score-weighted demographic and clinical characteristics of women who participated in HBCR and CBCR** Baseline demographic and clinical characteristics of women who participated in HBCR and CBCR within Kaiser Permanente Southern California women before and after propensity score weighting.

Measure (n = 753)	CBCR	HBCR	Overall	p-value	CBCR weighted	HBCR weighted
	(n = 371)	(n = 382)	(N = 753)		(n = 371)	(n = 382)
Age						
Mean (SD)	69.3 (11.7)	65.8 (11.8)	67.6 (11.9)	<0.001	66.5 (12.0)	65.8 (11.8)
Median (Min, Max)	70.3 (22.2, 93.0)	67.4 (30.5, 91.3)	69.1 (22.2, 93.0)			
Age by Group				<0.001		
Young Adult <45	13 (3.5 %)	22 (5.8 %)	35 (4.6 %)		(4.8 %)	(5.8 %)
Adult 45-64	92 (24.8 %)	137 (35.9 %)	229 (30.4 %)		(33.0 %)	(35.9 %)
Senior >64	266 (71.7 %)	223 (58.4 %)	489 (64.9 %)		(62.2 %)	(58.4 %)
Race/Ethnicity				0.461		
Non-Hispanic White	212 (57.1 %)	204 (53.4 %)	416 (55.2 %)		(53.4 %)	(53.4 %)
Non-Hispanic Black	31 (8.4 %)	46 (12.0 %)	77 (10.2 %)		(10.4 %)	(12.0 %)
Hispanic	90 (24.3 %)	97 (25.4 %)	187 (24.8 %)		(27.2 %)	(25.4 %)
AAPI (other)^a^	38 (10.2 %)	35 (9.2 %)	73 (9.7 %)		(9.1 %)	(9.2 %)
Marital Status				0.301		
Single	46 (12.4 %)	54 (14.1 %)	100 (13.3 %)		(14.4 %)	(14.1 %)
Married or Domestic Partner	217 (58.5 %)	202 (52.9 %)	419 (55.6 %)		(61.1 %)	(52.9 %)
Other	108 (29.1 %)	126 (33.0 %)	234 (31.1 %)		(24.5 %)	(33.0 %)
Language Spoken				0.349		
Non-English	32 (8.6 %)	26 (6.8 %)	58 (7.7 %)		(8.7 %)	(6.8 %)
English	339 (91.4 %)	356 (93.2 %)	695 (92.3 %)		(91.3 %)	(93.2 %)
Comorbidities						
Atrial Fibrillation	146 (39.4 %)	105 (27.5 %)	251 (33.3 %)	<0.001	(31.3 %)	(27.5 %)
Chronic Kidney Disease	114 (30.7 %)	109 (28.5 %)	223 (29.6 %)	0.510	(27.6 %)	(28.5 %)
COPD	98 (26.4 %)	93 (24.3 %)	191 (25.4 %)	0.514	(26.4 %)	(24.3 %)
Diabetes Mellitus	184 (49.6 %)	169 (44.2 %)	353 (46.9 %)	0.141	(49.5 %)	(44.2 %)
Hyperlipidemia	327 (88.1 %)	330 (86.4 %)	657 (87.3 %)	0.471	(86.5 %)	(86.4 %)
Hypertension	313 (84.4 %)	299 (78.3 %)	612 (81.3 %)	0.032	(82.1 %)	(78.3 %)
Heart Failure	209 (56.3 %)	202 (52.9 %)	411 (54.6 %)	0.341	(54.1 %)	(52.9 %)
Obesity	156 (42.0 %)	183 (47.9 %)	339 (45.0 %)	0.106	(52.6 %)	(47.9 %)
Prior Myocardial Infarction	164 (44.2 %)	201 (52.6 %)	365 (48.5 %)	0.021	(52.7 %)	(52.6 %)
Prior Stroke	80 (21.6 %)	53 (13.9 %)	133 (17.7 %)	0.006	(16.0 %)	(13.9 %)
Inpatient Stay^b^	311 (83.8 %)	283 (74.1 %)	594 (78.9 %)	0.001	(79.5 %)	(74.1 %)
BMI						
Mean (SD)	29.1 (7.1)	30.7 (6.3)	30.0 (6.8)	0.001	30.6 (6.4)	30.7 (6.3)
Median (Min, Max)	27.5 (18.1, 56.3)	29.6 (17.2, 54.4)	28.8 (17.2, 56.3)			
Charlson Comorbidity Index				0.177		
0-3	175 (47.2 %)	200 (52.4 %)	375 (49.8 %)		(51.7 %)	(52.4 %)
4+	196 (52.8 %)	182 (47.6 %)	378 (50.2 %)		(48.3 %)	(47.6 %)
Smoking Status				0.240		
Ever	126 (34.0 %)	138 (36.1 %)	264 (35.1 %)		(36.9 %)	(36.1 %)
Never (and unknown)	245 (66.0 %)	244 (63.9 %)	489 (64.9 %)		(63.1 %)	(63.9 %)
Referral Reason				<0.001		
Coronary Artery Disease^c^	112 (30.2 %)	169 (44.2 %)	281 (37.3 %)		(44.0 %)	(44.2 %)
CABG	82 (22.1 %)	53 (13.9 %)	135 (17.9 %)		(14.7 %)	(13.9 %)
CHF	54 (14.6 %)	83 (21.7 %)	137 (18.2 %)		(17.9 %)	(21.7 %)
VALVE	123 (33.2 %)	77 (20.2 %)	200 (26.6 %)		(23.4 %)	(20.2 %)
Distance to Nearest Medical Center						
Mean (SD)	10.5 (10.3)	12.9 (13.7)	11.7 (12.2)	0.008	10.6 (10.5)	12.9 (13.7)
Median (Min, Max)	7.71 (0.347, 92.9)	7.93 (0.343, 88.5)	7.77 (0.343, 92.9)			
Number of Sessions Completed						
Mean (SD)	20.2 (13.8)	24.7 (12.7)	22.5 (13.5)	<0.001	19.6 (14.0)	24.7 (12.7)
Median (Min, Max)	18.0 (2.00, 83.0)	25.0 (1.00, 49.0)	23.0 (1.00, 83.0)			

a The ethnic group noted as "AAPI (other)" includes Asian American, Pacific Islander, Native Alaskan, Other, and Unknown.

b Refers to study participants with an inpatient hospital stay prior to cardiac rehabilitation.

c The referral reason “coronary artery disease” includes patients referred to cardiac rehab for angina, myocardial infarction, and percutaneous coronary intervention.

In the current study, we found that compared to CBCR, participation in a technology-enabled HBCR program was associated with similar 12-month all-cause hospitalizations in women. These results are consistent with prior randomized controlled trials comparing home-based and center-based cardiac rehabilitation [1]. Our study is novel for several reasons.

To our knowledge, this study is the largest single-study sample of women participating in HBCR. The most recent Cochrane metanalysis comparing outcomes between HBCR and CBCR, which included 23 randomized controlled trials conducted between 1984 and 2016, reported a cumulative population of 549 women (with four trials omitting women altogether) [1].

Additionally, our study is the first to include a large population of demographically diverse and medically complex patients. The overall study population included 45 % non-white populations (25 % Hispanic, 10 % non-Hispanic Black, and 10 % Asian American, Pacific Islander, Native Alaskan, or other race). With regards to race/ethnicity, most of the Cochrane metanalysis trials (n = 19/23) did not report race/ethnicity and among the four trials that did report race/ethnicity, the study populations were all predominantly white [1]. With respect to medical complexity, we used the Charlson comorbidity index (CCI) to evaluate patients’ risk profiles. This index is a widely used indicator of 12-month mortality risk, previously validated among patients with cardiovascular disease [20,21]. Half the patients (50.2 %) in this study had CCI≥4, suggestive of moderate-high risk of 12-month mortality-an additional novel finding in light of the fact that majority of prior studies on focused on low-risk patient populations.

Our study is also the first report on hospitalizations as the primary outcome-a clinical outcome that is distressing for patient and families and costly for health systems. This is in contrast to the limited women-only CR literature there has focused on surrogate clinical outcomes such as cardiorespiratory fitness [22,23], changes in clinical risk factors [22], and changes in psychosocial well-being [22].

Finally, we found that women in the HBCR cohort lived farther from their nearest medical center and completed more cardiac rehabilitation sessions as compared to their CBCR counterparts. These differences are important for several reasons. Several studies have established that cardiac enrollment and adherence is significantly lower in women compared to men, with an inability to travel established as a common reason for lack of participation in CBCR [13,15].

This study is not without limitations, which should be considered when interpreting the results. The retrospective observational study design necessitated the use of statistical techniques to mitigate the impact of known confounding variables. However, it is impossible to account for all potential confounding variables, including those that may not be documented in the available data. Second, the data analyzed in this study occurred prior to the onset of the COVID-19 pandemic. This deliberate choice was made to eliminate the potential influence of pandemic-induced healthcare system changes that would have affected our primary outcome (12-month hospitalizations). Future studies assessing HBCR in women should analyze outcomes in the post-COVID-19 pandemic period.

Finally, our study population was composed of women who receive their healthcare within the integrated healthcare system of Kaiser Permanente Southern California (KPSC), where patients receive coordinated care delivered across various domains, including hospitals, outpatient medical office buildings and KPSC pharmacies that all utilize a single electronic medical record to co-ordinate care. This integrated health care delivery system may result in a population of women who are healthier at baseline compared to the general population of women, potentially limiting the generalizability of our findings to women receiving care with less integration or women who are uninsured or under insured.

Strengths of the current study include the use of a large, diverse well characterized population of KPSC patients with comprehensive electronic health records and pharmacy records to examine baseline comorbidities, adherence to cardiac rehabilitation, and 12-month clinical outcomes.

In conclusion, in this large and demographically diverse women-only CR cohort study that included medically complex women, we found that a technology-enabled HBCR program is a viable alternative to traditional CBCR. Further studies are needed to prospectively validate our findings.

## Data availability

The data analyzed in this study were accessed through a Data Use Agreement and under Institutional Review Board approval, thus, they are not publicly available. Due to the sensitive nature of data, anonymized data that support the findings of this study may be provided upon reasonable request, with permission and established agreement with the data provider, and after the legal and ethical reviews on reasonable request from qualified researchers with documented evidence of human subjects protection training.

## CRediT authorship contribution statement

**Michael Najem:** Conceptualization, Writing – review & editing. **Mark Duggan:** Methodology, Software, Formal analysis. **Rebecca Gambatese:** Methodology, Project administration, Data curation. **Rebecca Hill:** Formal analysis. **Su-Jau Yang:** Methodology, Data curation, Formal analysis. **Columbus Batiste:** Conceptualization. **Tadashi Funahashi:** Conceptualization. **Chileshe Nkonde-Price:** Conceptualization, Writing – review & editing, Supervision.

## Declaration of competing interest

No conflicts of interest exist.
